# Chagas Disease Control—Many Approaches to Prospect

**DOI:** 10.3390/tropicalmed8080395

**Published:** 2023-08-02

**Authors:** Marta H. Branquinha, Leandro S. Sangenito, Simone S. C. Oliveira, Claudia M. d’Avila-Levy, André L. S. Santos

**Affiliations:** 1 Laboratório de Estudos Avancados de Microrganismos Emergentes e Resistentes (LEAMER), Departamento de Microbiologia Geral, Instituto de Microbiologia Paulo de Goes, Universidade Federal do Rio de Janeiro, Rio de Janeiro 21941-902, Brazil; 2 Instituto Federal de Educação, Ciência e Tecnologia do Rio de Janeiro, Nilópolis 26530-060, Brazil; 3 Laboratório de Estudos Integrados em Protozoologia, Instituto Oswaldo Cruz, Fundação Oswaldo Cruz (FIOCRUZ), Rio de Janeiro 21040-360, Brazil; 4 Programa de Pós-Graduação em Bioquímica, Instituto de Química, Universidade Federal do Rio de Janeiro, Rio de Janeiro 21941-909, Brazil

Chagas disease is an emerging and neglected tropical disease caused by the protozoan parasite *Trypanosoma cruzi,* estimated to infect 8 to 10 million people worldwide, according to the World Health Organization [1]. This illness was discovered by Carlos Chagas in 1909 in Brazil [2], and more than a century later, it remains a challenge for humankind. One of the reasons for this scenario is the fact that, as a neglected disease, it receives very little financial support, with most investments concentrated on basic research and drug development [3].

Besides the epidemiological relevance of Chagas disease in Latin America, where it is endemic in 21 countries, the infection is a public health concern in many countries worldwide due to immigration, leading to significant economic burden on healthcare systems [1,3,4]. The parasite is transmitted by insect vectors belonging to the Triatominae family to vertebrate hosts, including humans and many different wild and domestic animals, which makes transmission control a necessity [4]. In addition, other transmission routes can be described, including blood transfusion and congenital infection, and more recently, oral transmission is gaining importance [4]. These aspects must be considered when considering the control of Chagas disease. In this sense, interventions to interrupt vector transmission coupled with serological blood-bank screening in many Latin American countries have improved the epidemiological aspects of the disease in the last 30 years [5]. However, the continuation of these public health programs is needed to maintain success [3].

This gains greater importance when the chemotherapy field is studied. In this regard, treatment options are currently limited to benznidazole and nifurtimox, as have been used for several decades, resulting in the selection of resistant strains [6]. In addition, both compounds display questionable efficacy and high toxicity and act mainly in the acute phase of the disease, which is usually mild or non-symptomatic [6]. However, the chronic phase that occurs many years after infection is responsible for severe manifestations, such as cardiomyopathies and digestive complications, leading to disability and higher mortality rates [7], demands novel treatment options. 

In this context, different approaches were addressed in this Special Issue relating to Chagas disease control. In the diagnosis field, Rivadeneira-Barreiro and co-workers [8] focused on the study of *T. cruzi* acute phase proteins in infected dogs in a coastal town in Ecuador as a means for the diagnosis, monitoring and prognosis of the parasite infection, implying that reduced paraoxonase-1 levels are suggestive of an oxidative stress response in seroreactive animals with no evident signs of inflammation. The research group of Simone Kann and collaborators [9] showed that electrocardiogram alterations, although nonspecific, can be considered an early indicator in Chagas disease screening, as detected in indigenous populations in the Sierra Nevada de Santa Marta, in Colombia, leading to the early treatment of the disease. Finally, Mary Lynn and colleagues [10] contributed a paper describing the urgent need for pregnancy screening for Chagas disease in western El Salvador due to the high rate of neonatal complications, reinforcing the growing concern related to the congenital form of the disease.

This Special Issue explores the vector’s contribution to disease spread. Velásquez-Ortiz and co-workers [11] detected high parasite loads in triatomines belonging to 10 species in different regions in Colombia, including species whose role as vectors is still unknown. The results pointed to the possible role of secondary species in *T. cruzi* transmission and the need to improve vector control programs in the country. Oliveira-Correia and collaborators [12] reinforced this approach by pointing to the relevance of the correct taxonomic identification of triatomines in South America due to the distinct epidemiological importance of each species and proposing the characterization of external female genitalia as a reliable technique in the identification of *Triatoma* species. In addition, Bates and colleagues [13] highlighted the importance of assisting community members in home reconstruction projects in rural areas to prevent Chagas disease by reducing exposure to the vectors, and the study provided evidence that project and social facilitators are more prone to help reconstruction despite the low personal economy. As a proposal to insecticide-resistant *Triatoma infestans* control, the major Chagas disease vector in southern Latin America, Baldiviezo and co-workers [14] studied the use of an alginate-based microencapsulated formulation of the entomopathogenic fungus *Beauveria bassiana.* The results showed the feasibility of the bioinsecticide to reduce vector transmission.

Concerning chemotherapy, Silva-Oliveira and co-workers [15] determined the effects of two aminopyridine derivatives complexed with Cu^2+^ against *T. cruzi* trypomastigote forms. Both compounds affected important cellular structures, such as the plasma membrane and the mitochondrion, besides the inhibition of the association index with LLC-MK_2_ cells. In this regard, these molecules may be candidates for antiparasitic drug development. Finally, a comprehensive review by De Fuentes-Vicente and collaborators [16] intends to provide an up-to-date pool of information on Chagas disease and the current research trend in order to provide insights into the search for more effective control approaches.

In summary, coordinated strategies involving proper diagnosis, vector control, reduced congenital infection and blood transmission, in addition to the development of efficacious anti-*T. cruzi* drugs, must be adopted to ensure the prevention of Chagas disease transmission. The editors hope that this Special Issue stimulates the distinct fields involved in the continuing efforts in the search for novel strategies that may lead to the improvement in Chagas disease control.
